# Biomarkers as targets for CAR-T/NK cell therapy in AML

**DOI:** 10.1186/s40364-023-00501-9

**Published:** 2023-06-17

**Authors:** Ruonan Shao, Zijian Li, Honglei Xin, Suyu Jiang, Yilin Zhu, Jingan Liu, Rong Huang, Kailin Xu, Xiaofeng Shi

**Affiliations:** 1 Second Affiliated Hospital of Nanjing Medical University, No.262, North Zhongshan Road, Nanjing, 210003 Jiangsu China; 2 Affiliated Hospital of Xuzhou Medical University, Xuzhou, 221004 Jiangsu China

**Keywords:** Acute myeloid leukemia, AML, CAR-T, CAR-NK, Target, Treatment, Research progress

## Abstract

The most common kind of acute leukemia in adults is acute myeloid leukemia (AML), which is often treated with induction chemotherapy regimens followed by consolidation or allogeneic hematopoietic stem cell transplantation (HSCT). However, some patients continue to develop relapsed or refractory AML (R/R-AML). Small molecular targeted drugs require long-time administration. Not all the patients hold molecular targets. Novel medicines are therefore needed to enhance treatment outcomes. T cells and natural killer (NK) cells engineered with chimeric antigen receptors (CARs) that target antigens associated with AML have recently been produced and are currently being tested in both pre-clinical and clinical settings. This review provides an overview of CAR-T/NK treatments for AML.

## Background

Blocking differentiation and boosting proliferation are hallmarks of acute myeloid leukemia (AML), a clonal malignant hematologic illness. The "3 + 7" regimen of anthracycline and cytarabine is the gold standard treatment for AML, after which high-dose cytarabine or hematopoietic stem cell transplantation (HSCT) may be considered [1]. The 5-year overall survival (OS) is low for individuals with relapsed or refractory (R/R) AML or those who are unsuited for HSCT [2]. Novel small molecular targeting drugs, such as gilteritinib (FLT3 inhibitor) [3], enasidenib (IDH2 inhibitor) [4], ivosidenib (IDH1 inhibitor) [5], or venetoclax (BCL-2 inhibitor) [6], are promising for these patients. In spite of this, many cases of R/R AML persist that do not have a mutation in FLT3, IDH1, or IDH2. Nevertheless, most patients ultimately die from recurrence or disease progression due to treatment resistance or harmful side effects. Because of this, research into potential novel therapies for AML is essential.

Chimeric antigen receptor (CAR)T cells and natural killer (NK) cells are engineered cell treatments created to identify and kill tumor cells. The ability of both types of cells to recognize and bind tumor targets is based on the same principle of receptor-ligand binding, but the effector functions they exert upon binding are different.

CAR-T cells are engineered to express a chimeric antigen receptor and other specialized molecules that allow them to identify, bind to, and destroy their intended targets. When CAR-T cells are infused directly into a patient's bloodstream, they kill cancer cells without causing as many systemic, non-specific adverse effects as traditional anti-tumor treatments [7].

NK cells, just like T cells, can also be engineered to express the same CAR. CAR-NK cells, just like CAR-T cells, can recognize and target cancerous cells [8]. The natural characteristics of CAR-NK cells, however, give them a number of benefits over CAR-T cells, including their immediate availability, inducible proliferation, longer lifespan, and capacity to generate cytokines and chemokines which increase during infection [9]. Additionally, when compared to CAR-T cells, the safety profile of CAR-NK cells is much more reassuring. Nonetheless, there is still a small chance of developing graft-versus-host disease (GVHD) when employing donor NK cells [10].

In addition to Kymriah and Yescarta, two more CD19 CAR-T cell products, Tecartus (also known as Brexucabtagene Autoleucel or KTE-X19) from Gilead and Breyanzi (also known as lisocabtagene maraleucel, liso-cel) from Bristol-Myers Squibb, have been approved by the FDA for the treatment of B-cell malignant hematological disorders [11]. Two BCMA CAR-T cell products, Abecma (idecabtagene vicleucel) and Carvykti (ciltacabtagene autoleucel) also are approved, recently. This establishes a benchmark for the development of novel AML therapeutics. If targets as successful as CD19, or BCMA are found, AML immunotherapy with CAR may achieve a comparable outcome. The efficacy of CAR-T and CAR-NK cells in the treatment of AML has been demonstrated in a number of in-vitro and in-vivo investigations [7, 9].

This review primarily focuses on target selection and the corresponding research status of CAR-T therapy for AML, while also taking into account current interest in NK cells in relation to those targets. The detailed properties and distributions of the targets are described in Table 1.

**Table 1:** Introduction to the targets involved in the study of CAR-T/NK for AML

Targets	Molecule type	Function	Current recruiting research		Distribution						References
					**Normal**	**Disease status**		**HSC**	**LSC**	**AML primitive cells**	
**CD33 (Siglec-3)**	Sialic acid-binding immunoglobulin, adhesion molecule	Myeloid differentiation antigens with endocytic properties, sialic acid-dependent cell adhesion molecules	Phase I/II clinical trials on CAR-T in China and US Phase I clinical trials on CAR-NK in China and US Phase I clinical trials on CD33/CLL1 CAR-T in China A phase I clinical trial on CD33/CLL1 CAR-NK in China		Myeloid cells (including myeloid precursor cells), neutrophils, NK cells, B cells, hepatic Kupffer cells, and microglia in the central nervous system	Expressed in approximately 85–90% of AML patients and in nearly 90% of AML cells in patients, has greater expression in normal karyotype or NPM1-positive AML		Yes	Yes	Yes	[12–14]
**CD123 (IL3Rα)**	Type I cytokine receptor	IL-3 receptor	A phase I clinical trial on CAR-NK in China Phase I/II clinical trials on CAR-T in China, US and Germany		Myeloid cells (including myeloid precursor cells), dendritic cells, endothelial cells, basophils, trachea, gastrointestinal tissue	Expressed abundantly on 78% of AML primary cells, LSC		Yes	Yes	Yes	[15–18]
**Siglec-6**	Sialic acid-binding immunoglobulin-like agglutinin 6	Immunosuppressive molecules	A phase I/II clinical trial on CAR-T in China		B cells, mast cells, placenta	Usually expressed in leukemia patients, including 60% of AML primary cells and stem cells		No	Yes	Yes	[19]
**TIM3 (HAVCR2)**	T-cell immunoglobulin and mucin structural domain 3, immunoglobulin superfamily	Immunomodulation through regulation of macrophage activity, inhibition of Th1 and Th17 effects and attenuation of TCR signaling; the synthesis of TIM3 to lessen the immune system's cytotoxic killing effect is one of the immunological escape strategies in AML	Preclinical trials: no current clinical trials using TIM3 CAR-T/NK		T cells, myeloid cells, NK cells, lung tissue	Expressed in only 6% of AML patients		No	Yes	Yes	[20]
**CD7**	Transmembrane glycoproteins, belonging to the immunoglobulin superfamily	Functions as a co-stimulatory receptor for T and B cell interactions during lymphocyte development, but function may be redundant	Phase I/II clinical trials on CAR-T in China		Activated T cells, NK cells, and some lymphoid and myeloid precursor cells	Expressed in 30% of adult AML patients		No (or transiently expressed)	Yes	Yes	[21]
**CD70**	Tumor necrosis factor receptor ligand for CD27, type II transmembrane glycoprotein	Tumor necrosis factor receptor	An early phase I clinical trial on CAR-T in China		Dendritic cells, B cells, upregulated on activated immune cells, monocytes and regulatory T cells expressed most	Overexpressed on solid tumors, lymphatic and myeloid tumors (including AML)		No	Yes	Yes	[22, 23]
**ILT3 (LILRB4)**	Immunoglobulin-like receptor B	Inhibition of T cell activation and proliferation; ILT3 expression on AML cells is thought to be used to bypass immune surveillance, particularly in the myeloid subtype		An early phase I clinical trial on CAR-T in China	Myeloid antigen presenting cells (including monocytes)	AML cells		No	Yes	Yes	[24, 25]
**NKG2D ligand (NKG2DL)**	NKG2D is a natural killer cell surface activating receptor (natural killer cell group 2 member D), a C-type lectin-like receptor protein	Activating receptors		Phase I clinical trials on CAR-NK in China and US	NKG2D ligand expression is limited in healthy tissues, mainly in NK cells, γδ T cells, CD8^+^ T lymphocytes, some CD4^+^ T cell subsets, Treg cells, endothelial cells, myeloid-derived suppressor cells	Upregulated in response to DNA damage, severe or malignant transformation; detected in hematological and solid tumors but is lowly expressed		No	Yes	Yes	[26–30]
**CD276 (B7-H3)**	Transmembrane protein, a co-receptor belonging to the B7 family of immune checkpoint molecules	Immune checkpoint molecules		A clinical trial on CAR-T in China	Antigen presenting cells, HSCs (low expression)	Overexpression on primary AML cells, especially monocyte subtype; expressed in 39–80% of bone marrow samples from AML patients		Yes(low)	Unknown	Yes	[31]
**CD117 (c-kit)**	Homologous receptor for stem cell factor (c-kit), type III tyrosine kinase receptor	Mast cell/stem cell growth factor receptor, which plays an important role in maintaining HSC homeostasis		The only one clinical trial on CAR-T in China was terminated because the therapeutic effect was not as expected	Gonadal, myeloid, erythroid precursor cells, HSCs, mast cells, melanocytes, Cajal mesenchymal cells, endothelial cells (e.g., skin, breast tissue)	Expressed on 80–90% of AML primary cells		Yes(high)	No	Yes	[32]
**FLT3 (CD135)**	Fms-related receptor tyrosine kinase 3, type III cytokine receptor	Maintenance of normal hematopoietic stem cell and precursor cell functions, including proliferation and differentiation. Presence of FLT3 mutation indicates poor prognosis		Phase I/II clinical trials on CAR-T in China	CNS, small intestine, testis, HSCs	Expressed on 54–90% of AML primary cells		Yes	Yes(high)	Yes	[18, 33–35]
**CD19**	Transmembrane proteins	Facilitates survival of B-cell development		A phase II/III clinical trial on CAR-T in Israel A phase I/II clinical trial on CAR-T in China	Prevalent in all stages of B cells except HSCs and plasma cells	There is a strong correlation between abnormal CD19 expression and t (8;21) in AML		No	Unknown	Yes	[36]
**CD174 (Lewis-Y, LeY)**	Sphingolipids (diatomic glycosyltransferases)	Blood group antigens		A phase I clinical trial on CAR-T in Australia but with unknown status	Limited expression in normal tissues, mainly in small intestinal endothelial cells	Expressed in a wide range of solid and blood tumors		Yes	Possible	Yes	[37–39]
**CLL-1 (CLEC12A)**	Type II membrane glycoprotein, C-type lectin-like molecule	Inhibitory receptors, involved in maintaining immune homeostasis		Phase I/II clinical trials on CAR-T in China and US Phase I clinical trials on CD33/CLL1 CAR-T in China An early Phase I clinical trial on CD33/CLL1 CAR-NK in China	Myeloid cells, myeloid precursor cells, lung tissue, gastrointestinal endothelial cells	Expressed on 78–92% of AML primary cells		Yes	Yes	Yes	[40, 41]
**CD38**	Glycoproteins	Cyclic ADP-ribose hydrolase		Phase I/II clinical trials on CAR-T in China	NK cells, B cells, Plasma cells, HSCs (low expression)	Highly expressed on primary AML cells and plasma cells from multiple myeloma		Yes(low)	No	Yes	[42–44]
**CD44v6**	Glycoproteins	Intercellular, cell–matrix adhesion receptors		A phase I/II clinical trial on CAR-T in Czechia and Italy was terminated due to the lack of target’s expression and low patient recruitment	Widespread expression on multiple tissue types, activated T cells, monocytes, keratinocytes, but all expressed at low levels	Expression present in 60% of AML patients, relatively tumor specific		No	Unknown	Yes	[45]
**FRβ**	Folate binding protein receptor	Assist with folic acid intake		Preclinical trials: no current clinical trials using FRβ CAR-T/NK	Myeloid cells, HSCs (low expression), activated macrophages	Expressed on 70% of AML primary cells, also upregulated with ATRA, HDAC		No	Yes	Yes	[46, 47]
**GM-CSF (CD116/CD131)**	Granulocyte–macrophage colony-stimulating factor	Granulocyte–macrophage colony-stimulating factor		Preclinical trials: no current clinical trials using GM-CSF CAR-T/NK	Myeloid cells	CD116 is expressed in 63–78% of AML patients, especially those with FLT3 mutations		Unknown	No	Yes	[48]
**CD25 (IL-2Rα)**	Type I transmembrane protein	Unknown		Only used in ADC studies in phase I/II clinical trials. No current clinical trials using CD25 CAR-T/NK	T, B cells, thymocytes, myeloid precursor cells, oligodendrocytes	found in 25% of patients with AML		No	Yes	Yes	[49–51]
**CD32**	Immunoglobulin superfamily, glycoprotein	Unknown		No current clinical trials using CD32 CAR-T/NK	Monocytes, granulocytes, B cells, macrophages, platelets	Approximately 34% of people with AML express it		No	Yes	Yes	[52]
**CD47**	Immunoglobulin superfamily, glycoprotein	Binds SIRPa and inhibits cytophagy		Used in monoclonal antibody studies in phase I-III clinical trials. No current clinical trials using CD47 CAR-T/NK	Keratinocytes, immune cells	Expressed in almost all AML patients		Yes(low)	Yes	Yes	[53]
**CD56**	Neuronal cell adhesion molecule, glycoprotein	Unknown		A phase I/II clinical trial on CAR-T in China but was unknown status A clinical trial on CAR-T in China was terminated with unknown reasons	Keratinocytes, NK, T cells, nerve, neuroendocrine tissue	Expressed on AML primary cells and possibly on LSCs		No	Possible	Yes	[54]
**CD90 (Thy1)**	Immunoglobulin superfamily, glycoprotein	Unknown		No current clinical trials using CD90 CAR-T/NK	Endothelial cells, neural tissue, thymocytes		Expressed on AML primary cells and possibly on LSCs	Yes	Possible	Yes	[55]
**CD96**	Type I transmembrane proteins, immunoglobulin superfamily	Modifications in T cell and natural killer cell adhesion		No current clinical trials using CD96 CAR-T/NK	T cells, NK cells, endothelial cells		Expressed on LSC, AML primary cells	Yes(low)	Yes	Yes	[56]
**IL1RAP**	Auxiliary proteins	IL-1 receptor		A clinical trial on CAR-T in France	Liver, esophagus, gastrointestinal tissues, genitourinary tract tissues		Expressed on 80% of AML primary cells	No	Yes	Yes	[57]
**MUC1**	Glycoproteins	Mucosal protective effect, transmitting cellular signals		A phase I/II clinical trial on CAR-T in China but was unknown status	Expressed on most general epithelial cells and Treg cells, lymphoid tissue, lung endothelial cells, gastrointestinal endothelial cells, genitourinary tissues, placenta, skin		Expressed on LSC, AML primary cells	Yes(low)	Yes	Yes	[58]
**WT1**	Intracellular peptides, zinc finger DNA binding proteins	Endogenous antigens, transcription factors		A phase I clinical trial on CAR-T in China	Kidney, oviduct, endothelium, testis		Expressed on AML primary cells, LSC	Yes(low)	Yes	Yes	[59–61]
**PR1/HLA-A2 (h8F4)**	Intracellular peptide. PR1 peptide derived from leukemia-associated antigen protease 3 and neutrophil elastase	Endogenous antigens		Preclinical trials: CAR-T targeting either PR1 or HLA-A2 have been shown to be effective in eliminating AML primary cells in in vitro trials. No current clinical trials using PR1/HLA-A2 CAR-T/NK	PR1 is present in primitive asplenophilic granules of neutrophils and HLA-A2 is overexpressed on primitive cells of the myeloid lineage		Expressed on AML primary cells	Yes	Yes	Yes	[62]
**CD93**	C-type lectin transmembrane receptor	Cell adhesion, host defense		Used in preclinical trials and vaccine trials. No current clinical trials using CD93 CAR-T/NK	Expressed on neutrophils, monocytes, mature myeloid cells, endothelial cells, but not on HSCs, other myeloid precursor cells, erythrocytes, TB lymphocytes, platelets		Expressed on AML primary cells	No	Unknown	Yes	[63]
**PD1**	PD1 is a member of the co-stimulatory receptor B7/CD28 family	When PD1 binds to its programmed death ligand 1 (PD-L1), it controls T-cell activation, stops T-cells from multiplying and making a lot of IFN-, TNF-, and IL-2, and makes it harder for T-cells to live. cTLA-4 also controls T-cell activation in a bad way		Used in monoclonal antibody studies in phase I/II clinical trials. No current clinical trials using PD1 CAR-T/NK	Activated T lymphocytes express PD-1 on their surface, in addition to other cells (e.g., B cells, NK cells, dendritic cells, macrophages, vascular endothelial cells, epithelial cells)		Upregulated expression of PD1 on the surface of bone marrow CD8^+^ T cells in AML patients	Unknown	Unknown	Yes	[64]
**PRAME**	Intracellular peptides	Testicular tumor antigen		Used in TCR T-cell studies and vaccine trials in phase I/II clinical trials No current clinical trials using PRAME CAR-T/NK	CD8^+^ T cells		Expressed on 41–55% of AML primary cells	Yes(low)	Yes	Yes	[61]
**mLPA**	Methyl-hemolytic phosphatidic acid	CD1c-restricted T-cell antigen		No current clinical trials using mLPA CAR-T/NK	monocytes, dendritic cells, TB lymphocytes		Found on AML primary cells, LSCs	No	Yes	Yes	[65]
**IDH1 (R132)**	Isocitrate dehydrogenase 1, intracellular	Glycolic acid bypass, tricarboxylic acid cycle		Due to the intracellular presence of the antigen, it is temporarily impossible to attach it to the CAR. So, there is no current clinical trials using IDH1 CAR-T/NK	Hepatocytes, cytotrophoblasts		Expressed on AML primary cells, LSCs; in primary AML patients, expression is present in approximately 20% of patients	Yes	Yes	Yes	[66]
**IDH2 (R140)**	Isocitrate dehydrogenase 2, intracellular	Glycolic acid bypass, tricarboxylic acid cycle		Due to the intracellular presence of the antigen, it is temporarily impossible to attach it to the CAR. So, there is no current clinical trials using IDH2 CAR-T/NK	Distal renal tubular cells, cytotrophoblasts		Expressed on AML primary cells, LSCs; about 20% of primary AML patients	Yes	Yes	Yes	[4, 67]
**NPM1mut**	Ribophilin 1 mutant	Ribosome biosynthesis, host-virus interactions		No current clinical trials using NPM1mut CAR-T/NK	Reduced specificity of cells and tissues		AML primary cells and LSCs express it	No	Yes	Yes	[68, 69]
**NOTCH2**	NOTCH signaling molecule isoform 2	Disease progression		No current clinical trials using NOTCH2 CAR-T/NK	Non-germline precursor cells, Paneth cells		Found in both primary cells and LSCs of AML	Yes	Yes	Yes	[70]
**PRL3**	Protein tyrosine phosphatase type Iva member 3	Enhanced PI3K/Akt signaling activation		No current clinical trials using PRL3 CAR-T/NK	Cardiomyocytes, neutrophils, atypical monocytes		Expressed on AML primary cells, LSC	Yes	Yes	Yes	[61]
**IL12RB1**	IL-12 receptor beta 1	Transmitting cytokine signals		No current clinical trials using IL12RB1 CAR-T/NK	T-cells, Kupffer cells, B-cells		Expressed on AML primary cells, LSC	Yes	Yes	Yes	[71]
**CD244/2B4**	NK cell activating/inhibitory receptor	NK cell-activated/inhibitory receptors		No current clinical trials using CD244/2B4 CAR-T/NK	Unknown		Expressed on both AML primary cells, LSC	Yes(high)	Yes(high)	Yes(high)	[72]
**RHAMM**	Intracellular peptides	Cellular matrix interactions		Due to the intracellular presence of the antigen, it is temporarily impossible to attach it to the CAR. So, there is no current clinical trials using RHAMM CAR-T/NK	Colonic		Expressed in AML primary cells, possibly in LSCs	Yes	Possible	Yes	[73]
**Survivin**	Intracellular peptides	Anti-apoptotic proteins (associated with embryogenesis)		Used in vaccine test. It is temporarily unable to attach antigen to CAR, so there are no current clinical trials using Survivin CAR-T/NK	Endothelial cells		Expressed on AML primary cells, LSC	Yes	Yes	Yes	[74, 75]
**hTERT**	Intracellular peptides	Telomerase complex subunit		Used in vaccine test. It is temporarily unable to attach antigen to CAR, so there are no current clinical trials using hTERT CAR-T/NK	Keratinocytes, testis, endothelial cells, placenta		Expressed in AML primary cells, possibly in LSC	Yes(low)	Possible	Yes	[76–78]
**CD4**	T lymphocyte membrane glycoprotein	Interaction with major histocompatibility complex class II antigens		No current clinical trials using CD4 CAR-T/NK are recruiting. But CD4 CAR-T is used in several phase I clinical trials for T cell malignancies or solid tumors in China and US	T-lymphocyte cells, and is expressed in nearly all T-cells		Expressed in 30%-40% of other AML subtypes, 65% of AML-M4, and 78.3% of AML-M5	No	Yes	Yes	[79]

*CAR-T* chimeric antigen receptor T cell, *AML* acute myeloid leukemia, *HSC* hematopoietic stem cells, *LSC* leukemic stem cells, *NK cells* natural killer cells, *IL* interleukin, *TCR* T cell receptor, *R/R* relapsed or refractory, *ATRA* all-trans retinoic acid, *HDAC* histone deacetylase, *ADC* antibody–drug conjugate, *SIRPα* signal regulatory protein alpha

## Targets that have entered clinical trials

As technology advances, more targets, for instance, CD123, CD33, CD70, FLT3, CD38, and many others, have entered clinical trials. In order to induce cell death in AML cells, these targets are selected based on their capacity to bind to surface receptors. As a result of promising findings in preclinical trials, these targets are now the subject of extensive research into their clinical pharmacology, human safety, and therapeutic efficacy.

### CD123

CD123, a high-affinity receptor for stem cell factor, is the α subunit of the interleukin 3 receptor (IL-3R). When its expression rises, AML patients' overall survival (OS) and progression-free survival (PFS) drop [15]. CD123 forms a heterodimer with β subunit of IL-3R to activate the JAK/STAT signaling which is required for hematopoiesis [80]. Furthermore, CD123 expression has been linked to a greater risk of treatment failure [81].

In AML patients, CD123 expression levels are correlated with the number of leukemic stem cells (LSCs) which are CD34^+^CD38^−^ quiescent cells and demonstrate chemotherapy resistance [82–84]. CD123 is a considerably more promising target than CD33 since it is highly expressed by AML and relatively less expressed by hematopoietic stem and progenitor cells [16]. Anti-CD123 CAR-T cells efficiently reduce the burden of leukemia in vivo with minimal damage to normal hematopoietic stem and progenitor cells (HSPCs) [85]. Despite its specificity, off-target still brings significant hematopietic toxicities, especially when adjuvants that increase CD123 expression on both AML and normal HSCs are added to improve targeted killing [86].

Using a panel of CD123-specific monoclonal antibodies, Thokala et al. engineered CD123 CAR-T cells with variable VL and VH chains (mAb) [87]. This type of CD123 CAR T cell exhibits potent cytotoxicity toward CD123^+^AML cells but much lower toxicity toward normal hematopoietic stem cells. Several researchers have proposed CAR-T123 as a new pretreatment regimen for producing remission and bridge to HSCT [86]. However, its low remission rate and the major complications, such as infection and hemorrhage when prolonging the transplantation interval, limit its usage. In this context, subsequent studies have sought to optimize protocols for CD123 CAR-T treatment. For instance, because CD123 is significantly expressed on endothelial cells as well as hematopoietic cells, CD123 CAR-T can accidentally injure these normal tissue cells, leading to endothelial cell damage and hematopoietic toxicity. In order to overcome this shortcoming, Nadia EI Khawanky et al. developed a third-generation CD123 CAR-T cell based on single-chain variable fragments (scFvs) and the CD28-OX40-CD3 intracellular signaling domains of humanized CSL362, which demonstrated anti-AML activity in xenograft mice without damaging epithelial tissue [17]. Furthermore, it was discovered that 5'-azacytidine therapy increased CD123 expression on leukemic cells, leading to the expansion of CD123 CAR-T cells and a boost in effector capacity, in addition to increased levels of tumor necrosis factor (TNF) production and elevated downstream phosphorylation of a key T cell activation molecule. This demonstrates the superior efficacy of combining co-stimulatory domains with the induction of antigen expression on leukemic cells, thus maximizing the killing effect while ensuring safe treatment.

Budde reported that, after CD123 CAR-T cell treatment, 3/6 individuals with R/R AML had complete remission (CR), and two obtained partial remission, while 66% of these patients subsequently receiving allo-HSCT [88] (NCT02159495). Despite the fact that CD123 CAR-T has been shown to be clinically effective, early trials showed that its efficacy was significantly lower than CD 19 CAR-T, likely due to low targeting specificity [85]. Whereas CAR-T123 with HSCT has shown promise for the treatment of AML, more study is needed to determine the regimen's long-term anti-leukemic efficacy, the ideal transplantation interval, and the prevention of graft-versus-host disease (GVHD).

CD123 CAR-T cell treatment caused long-term myelosuppression in individuals who did not receive subsequent allo-HSCT, so it is still an open question as to how to enhance CR rates and minimize potential hematopoietic toxicity. This highlights the need for novel clinical strategies that put the power of CAR-T technology under a dependable and quick “ON/OFF” switch that regulates when it is active and when it is turned off in order to lessen both short-term and long-term side effects. For instance, the Herpes simplex virus (HSV)-thymidine kinase suicide gene was one of the original safety switches that transformed the prodrug into a toxic compound, preventing further replication of cellular DNA [89]. As a result, cells expressing the suicide gene died after the prodrug was administered, but the immunogenicity of the enzyme after gene expression and the restrictions of the activation latency prevented rapid termination of the targeted killing effect. Walid Warda et al. used an inducible caspase 9 suicide gene safety switch to circumvent the masked resistance to target epitopes that can emerge on low antigen-expressing AML primary cells after interleukin-1 receptor (IL-1RAP) CAR-T treatment [90].

Other optimization techniques also can be used to generate controlled CAR-T effects. In 2020, the University of Pennsylvania ran a clinical experiment with mRNA electroporated CD123 CAR-T cells [91] (NCT02623582). Because of the potential for CAR-T cells to persist and cause myelosuppression if left in the body for too long, mRNA electroporation was favored over lentiviral transduction. In vivo activation of CAR-T cells was reported to be transient during the clinical trial, which was consistent with expectations. In 2021, Jan-Erik Meyer et al. developed UniCAR, a fast switchable universal CAR-T platform [92]. Specifically, the first element of the system is a universal CAR that does not interact with any human surface antigens but is recognized by a targeting module (TM) in the second part of the system. TM is the central component of such a platform, providing specificity towards predetermined cancer antigens. Due to the adaptability of the tumor-binding domain, it is able to target a wide variety of antigens in both solid tumors and hematological malignancies. The antigen-specific TM, such as TM123, can connect CAR to antigen, such as CD123, on the surface of myeloblastic cells, thus starting the killing. Notably, the short half-life of these TMs, which is below 30 min, allows for rapid deactivation of the UniCAR system by TM withdrawal, consequently avoiding the long-term harmful effects associated with the ongoing activation of CAR-T cells. The “ON/OFF” switch depends on TM administration or not. Moreover, preclinical data demonstrated that UniCAR-T cells lyse cancer cells at a lower TM dose compared to cytokine release induction, indicating a greater therapeutic window for clinical application. Preliminary results suggest CD123 UniCAR-T is well-tolerated and fast convertible with promising outcomes even at the lowest dose levels. The Phase 1A study is still recruiting patients (NCT04230265).

Targeting CD123 for anti-AML CAR treatment has seen the greatest research, with numerous ongoing clinical trials investigating its use in UniCARs [92](NCT04230265) and bispecific CARs [16](NCT04678336, NCT03766126, NCT02623582). It has been suggested that CD33 and CD123 can be targeted jointly in AML therapy because 70% of AML initial cells show both of these markers [16]. Several clinical trials exploring multiple antigens have been launched (NCT04010877, NCT04033302).

### CD33

Myeloid-specific transmembrane sialic acid-binding receptor CD33 is known to control leukocytes throughout the immune response due to its two extracellular Ig-like structural domains.

Initial feasibility studies showed that cytokine-induced killer cells could be used to deliver CD33-directed CAR T cells [93]. Myeloid-specific transmembrane sialic acid-binding receptor CD33 is known to control leukocytes throughout the immune response due to its two extracellular Ig-like structural domains. Almost all AML cells and leukemic stem cells express CD33 at high levels, whereas normal cells express it at low levels, even at low effector-to-target ratios of < 1:20, making CD33 a useful diagnostic marker and therapeutic target for AML. Recent research, however, has shown that CD33 is especially important since its loss from the surface of AML primitive cells as antigen-negative escape [93] is associated with a poorer prognosis for AML patients.

Ten individuals with R/R AML were enrolled in a phase I clinical research assessing the safety and efficacy of CD33 CAR-T cells [94] (NCT03126864). Three of these patients got cell treatment. Participants had adverse events such as cytokine release syndrome (CRS), immunological effector cell-associated neurotoxicity syndrome, tumor lysis syndrome, respiratory distress syndrome of grade 3, and infectious shock. Although no hepatotoxicity was recorded, all three recipients succumbed to the progression of the disease. In contrast, a case report of a 41-year-old male shows that CD33 CAR-T temporarily reduced the number of AML primitive cells in his bone marrow (NCT01864902), prior to disease progression 9 weeks after initiation of the treatment [95]. The therapy also caused CRS, indicating a partial response to the AML.

Preclinical and clinical research have frequently highlighted CD33’s potential as a target for the treatment of AML, but its use has not been standardized in AML patients until very recently. This is because its potent anti-AML activity is coupled with obvious side effects which may result from its expression on normal HSCs, albeit at low levels. Preclinical models have illustrated the typical hematopoietic toxicity associated with CD33-targeted treatments, such as hemocytopenia and decreased myeloid progenitor cells [96]. Severe hematopoietic suppression may lead to neutropenia and thrombocytopenia causing fatal infection and bleeding. This defect can be addressed in numerous ways. The first method is a combination of CD33 CAR-T treatment with CD33 knockout (KO) HSCs transplantation. In a recent study, Florence Borot et al. successfully used CRISPR/Cas9 to delete the CD33 antigen, and found no effect on the ability of HSCs to repopulate [96]. The dual-targeted guide RNA editing has also been proposed as an alternate method of CD33 modification. CD33 CAR-T cell treatment in combination with CD33 KO HSCs transplantation is likely to achieve precise targeting of AML cells and reduced myelotoxicity. This novel form of immunotherapy is attractive, but further research and clinical assessment are necessary prior to its application in humans. Furthermore, this approach may be extensible to other malignancies and antigens exhibiting analogous characteristics. The second strategy employs a "AND" logical gating strategy, whereby one CAR molecule is used for activation and another CAR molecule is used to provide co-stimulatory sites in the same T cell, optimizing the combination of antigen recognition to reduce "on-target/off-tumor" toxicity, and the killing effect of CAR-T can only be activated when at least two markers are expressed on the target cell [97]. While "OR" gating only requires the expression of one antigen in cells, "NOT" gating requires no other markers. BisCAR T cells which contain co-stimulatory domains of Nb 157/CD3ς and antiTIM3 scFv/CD28/4-1BB specifically killed CD13 + TIM3 + double positive tumors, but with reduced and likely acceptable toxicity to HSCs and other normal cells with only CD13 expression [97].

The third way is to select immortalized immune cell lines for CAR-T construction. Barsov et al*.* used these cells to produce CAR-T cells [98]. This approach is expected to lead to a commercially available cell therapy product, while avoiding manufacturing failure. However, irradiation of the immortalized cell line is essential before injection to avoid its malignant potential, compromising the in vivo durability of the cells. Additionally, to enhance on-target killing and reduce off-target toxicity, “AND” gating techniques may be employed to regulate CAR-T activity. Like the above mentioned CD123 UniCAR-T, logical gating solutions involve the employment of only one CAR molecule for activation and a second for co-stimulation. Additionally, other approaches, such as “ON” switch, “OFF” switch [99], SUPRA (split, universal, and programmable) CAR [100], RevCAR (Reverse CAR) [101], and STOP-CAR platform are also available [102]. Finally, maximizing the efficacy of CAR therapy requires optimizing techniques to enhance the production of CD33 targeted cells and decrease the time between single-cell collection and treatment [102].

The potential of bispecific CD33-CLL1 CARs [103] in clinical studies has brought into focus the necessity for rapid and efficient production methods to fully achieve this treatment's potential [103].

### CD7

The glycoprotein CD7 is a kind of transmembrane protein. CD7 is mostly expressed in T cells, NK cells, and their precursor cells in healthy people because it serves as a co-stimulatory receptor for T and B cell contacts throughout lymphocyte maturation [21, 104, 105]. Some myeloid progenitor cells in cord blood express it as well, though it is not clear what they are used for. CD7 is expressed in both normal and leukemic bone marrow precursor cells, suggesting that it may be a useful therapeutic target for AML treatment.

The first preclinical evidence released by the lab of Gomes-Silva on CD7 CAR-T indicates its therapeutic efficacy in AML [21]. However, CAR-T targeting CD7 may attack other T and NK cells, causing “fratricide”, and depleting the CAR-T cells after infusion. Conversely, CD7-deficient animals showed largely unchanged peripheral T cell function [21], which suggests that CD7’s role in mature T cells may be redundant. In light of these two considerations, it is possible that eliminating the CD7 gene from CAR-T cells may allow them to develop to their full potential, so achieving the intended therapeutic effect while reducing the risk of T-cell “self-mutilation” and maximizing the efficiency of CAR-anti-leukemia T cells. After using CRISPR-Cas9 technology to deplete CD7 levels from T cell, Silva et al. discovered that CD7 CAR-T cells had potent anti-AML efficacy in mice [105]. Eighty-five percent to ninety percent of CAR-T cells can have CD7 expression deleted using the CRISPR-Cas9 technique, with no discernible off-target effect in the therapy of AML. It is also possible to remove CD7 from the surface of cells in ways other than genome editing. CD7 can be eliminated by attaching to an scFv that is linked to the cell’s endoplasmic reticulum [106]. Thus, inhibiting CD7’s trafficking to the cell surface reduces its ability to “self-flagellate” CD7 CAR-T cells. However, substantial clinical trial comparisons of the long-term efficacy and feasibility of these two methods are required (NCT04762485, NCT04033302).

### CD70

CD70, a member of the tumor necrosis factor (TNF) family of type II transmembrane glycoproteins, is a ligand for the TNF receptor, CD27. It is significantly upregulated on AML cells, including LSCs. This makes CD70 an attractive therapeutic target for AML.

The anti-tumor benefits of modified CD70 CAR-T cells were demonstrated by Maus et al. at the 2021 annual meeting of the American Society of Hematology (ASH 2021) [106]. Moreover, CAR-T cells containing a truncated CD27, CD8 hinge and transmembrane region were found to be highly efficacious against AML. Additionally, LSCs can render more susceptible to CAR-T therapy by modulating their CD70 expression with hypomethylating agents and this was confirmed by Riether *et al* [22].

Patients with R/R AML are the focus of ongoing clinical trials with CD70 CAR-T cells (NCT04662294) and CD70 NK cells producing IL-15 (NCT05667155).

### ILT3 (LILRB4, CD85k)

ILT3, also known as leukocyte immunoglobulin-like receptor subfamily B member 4 (LILRB4), is an inhibitor of T cell activation and proliferation. Maintaining an immunosuppressive milieu for tumor cells, ILT3 may contribute to tumor’s evasion from the body’s immune system. ILT3 is expressed in myeloid cells and highly expressed in AML monocytes, therefore, representing an effective target for AML-M5.

In this regard, Samuel John et al*.* developed a ILT3 CAR-T therapy [24]. It was shown to trigger apoptosis specifically against monocytic AML in vitro and also decreased tumor burden in a xenograft model in vivo, with no observable adverse effects on normal hematopoiesis. To enhance ILT3 CAR therapeutic efficacy, it can be co-expressed with co-stimulatory domains or used in conjunction with other targeted therapies. Nevertheless, more work must be done to ensure the production of anti-ILT3 cells from scaled-up CAR-T to Good Manufacturing Practice grade in order to carefully address the various issues in production and application. These issues include the origin of the T cells, the limitations of the patient's own low lymphocyte count and the pollution of tumor cells when modifying autologous T cells, and the interference of HLA compatibility when infusing allogeneic T cells, etc. Clinical trials on ILT3 CAR-T are presently underway (NCT04803929).

### NKG2DL

NKG2DL, or natural killer group 2 member D ligand, is a tetrameric protein complex that, when attached to its receptor, can trigger cell death. It is composed of transmembrane proteins that are linked via glycosylphosphatidyl alcohols. NKG2DL is increased in response to genotoxic stress, infection by some pathogens, and, most notably, malignant transformation.

NKG2DL overexpression has been seen in a variety of solid and hematological cancers, including AML and multiple myeloma (MM) [26]. Evidence suggests that its expression levels are associated with significant predictive prognosis in many AML patients. Therefore, therapeutic applications for NKG2DL-targeting CAR-T cells may be extensive.

Preclinical models of ovarian cancer and MM have displayed anti-tumor effects of NKG2DL CARs [27]. Nonetheless, clinical trials involving similar targeted CAR-T cells in AML patients achieved limited success. Baumeister et al. did not observe any notable impact after a single infusion [28], while Sallman et al. administered multiple infusions at higher doses and reported that one out of 22 patients experienced morphologic leukemia-free state [29]. Furthermore, activation of NKG2DL already presented in T cells can lead T cells to self-collapse. About this matter, NKG2DL CAR-T cells can be used in combination with a phosphatidylinositol 3-kinase (PI3K) inhibitor [30], and the cells can be further modified to express short hairpin RNA to decrease the amount of NKG2DL on the surface of CAR-T cells. Two AML patients showed a partial response to the treatment.

Patients with AML, myelodysplastic syndrome (MDS), and MM participated in the first human clinical trial evaluating the safety and feasibility of NKG2DL CAR-T cells [28] (NCT02203825). This Phase I dose-escalation trial obtained adequate number of NKG2DL CAR-T cells using special collecting bags for isolation products, and larger containers to provide a larger surface area for cells to grow (such as G-Rex). The manufactured NKG2D-CAR T cells demonstrated functional activity against autologous tumor cells in vitro. However, the extent of T cell expansion, the proportion of CD4 to CD8 cells, and the transfection efficiency of CD8 + cells were highly variable after transduction, which might be related to the clinical heterogeneity of the patients, including ages, stage of diseases, and previous treatment regimens. Nonetheless, this clinical trial offers circumstantial evidence that it is possible to produce sufficient number and effects of NKG2D CAR-T cells in a short amount of time.

Increased ligand expression may also cause toxic side effects in ordinarily functioning tissues. In clinical trials, however, no dose-limiting toxicity, cytokine release syndrome, or CAR T cell-related neurotoxicity was observed, nor were there any significant autoimmune reactions or adverse events of grade 3 attributable to NKG2D-CAR T cells. NKG2DL CAR-NK and CAR-T cell therapies are now being tested in a number of active clinical trials (NCT05734898).

### FLT3

The FMS-like tyrosine kinase-3 (FLT3) receptor is one type of tyrosine kinase receptor. FLT3 ligand (FLT3Lg) is a natural ligand for FLT3. Upon FLT3Lg binds to FLT3, cells are activated. Furthermore, FLT3 mutations, which can be seen in 30% of AML patients, are extremely prevalent, making it one of the most frequently mutated genes [33]. The most prevalent FLT3 mutations are found in 24% of AML patients with internal tandem duplication (ITD) mutations and 7% of AML patients with tyrosine kinase structural domain (TKD) mutations [65, 100]. The PI3K/Akt, Raf/MEK/ERK, and JAK/STAT5 signaling pathways, which drive the progression of AML, are mediated by FLT3-ITD and FLT3-TKD. As a result, both FLT3-ITD and FLT3-TKD are linked to illness development and progression, and both indicate a bad prognosis.

Although FLT3 inhibitors, including gilteritinib [3] and midostaurin [107], have currently been used to improve clinical outcomes, allo-HSCT remains the only viable treatment option for patients with FLT3-mutated AML. Additionally, crenolanib, an FLT3 inhibitor, can upregulate FLT3-ITD expression on the cell surface, thus enhancing the targeting efficiency of FLT3 CAR-T [108].

Targeting a receptor can be accomplished in two ways, depending on its nature as a receptor. The first is to utilize anti-receptor antibodies, such as scFvs derived from anti-human FLT3 antibodies. Cesar Sommer et al. generated a kind of scFv-containing FLT3-CAR-T and examined its effects in a preclinical setting [109], and discovered that they removed primary AML primitive cells, as well as hematopoietic stem and progenitor cells, which bring certain bone marrow toxicity. This toxicity can be controlled by a way of rituximab-activated off-switches which deplete circulating CAR-T cells. Replication of natural binding ligands, FLT3L, is another approach for reducing immunogenicity and off-target toxic effects. Wang et al. developed FLT3L-4-1BB-CD3-CAR-T cells and evaluated their efficacy against FLT3^+^ leukemia cells [34]. FLT3L CAR-T cells demonstrated a greater lethality towards ITD-type mutations compared to wild-type FLT3, thereby protecting normal cells and hematopoiesis.

Several parallel clinical trials are currently enrolling patients (NCT05023707, NCT05445011, NCT05017883, and NCT05432401) to investigate the efficacy of FLT3 CAR-T cell therapy. Second-generation FLT3 CAR-T studies targeting FLT3 extracellular structural domain epitopes are in phase I clinical trials. One such study is using a kind of FLT3 CAR-T cells, named Amg553 (NCT03904069) which contains a scFv structural domain that binds extracellular epitopes of the FLT3, a CD28 co-stimulatory structure domain, and a CD3 zeta chain subunit activation structural domain. The preliminary results show that Amg553’s CAR-T cells target FLT3-expressing leukemia cells but spare normal hematopoietic stem cells, reducing therapeutic side effects. Additionally, Amg553’s CAR-T cells have a shorter lifespan, which allows for faster clearance and fewer side effects. Finally, Amg553’s CAR-T cells can be manufactured faster than other therapies, reducing treatment time. In summary, Amg553’s greater selectivity, safety, therapeutic efficacy, and shorter production time make it a promising treatment for acute myeloid leukemia.

### CLL-1 (CLEC12A, MICL, KLRL1, DCAL-2)

Despite being regarded as a type II transmembrane glycoprotein with an inhibitory receptor function, the ligand for C-type lectin-like molecule 1 (CLL-1), also known as CLEC12A, MICL, KLRL1, or DCAL-2, has not been determined. As CLL-1 is not expressed in healthy tissues, it presents a promising therapeutic target.

Zhang et al. reported the first successful treatment using CLL-1 CAR-T cells. Following the administration of CAR-T, the patient had grade I–II cytokine release syndrome (CRS), which was characterized by an increase in body temperature and brief hypotension [40]. On day 29, the patient experienced morphological CR and MRD negative, which persisted for almost 9 months (NCT00846703). Multiple preclinical investigations have supported the potent anti-leukemic action of CLL-1 CAR-T without interfering with normal HSC. Subsequent modifications focused on enhancing the effect of the therapy, including improvement of the CAR-T construct as well as simultaneous transgenic expression of cytokines. However, these adjustments can lead to increased cytotoxicity. To address this challenge, Tashiro et al. developed a Caspase-9 suicide gene system [41] to provide targeted killing with the potential to modify its action.

Numerous CAR-T cell clinical trials targeting CLL-1 are currently recruiting participants (NCT05252572, NCT4219163, NCT04884984).

### CD38

CD38 is a glycoprotein that is overexpressed in hematological malignant cells and broadly expressed on immune cells (especially plasma cells) and erythrocytes but not on HSCs [42, 110]. This expression feature allows CD38 as a therapeutic target. Myeloma patients have responded favorably to daratumumab, an anti-CD38 monoclonal antibody [111].

CD38 has a particular significance in AML, given that up to 83% of AML cells express CD38. Efforts must, therefore, be made to maximize the level of CD38 expression and binding intensity for these CAR-T treatments. To further enhance the efficacy of CAR-T therapy in AML, a study by Kazuyoshi Yoshida showed that all-trans retinoic acid (ATRA) could raise CD38 expression on AML cells [43].

In order to prevent CAR-T/NK cell depletion, CD38 expression should be knocked down from the T or NK cells using, for example, the CRISPR-Cas9 technology [44]. CD38 is expressed in immune cells such as T, B, and NK cells. Anti-CD38 CAR-NK cells with CD38 knockdown have shown significant benefits in preclinical studies [42]. Six AML patients who suffered from recurrent AML following allo-HSCT have been enrolled in CD38 CAR-T clinical studies (NCT04351022). Four weeks after the initial CD38 CAR-T cell infusion, four out of the six patients achieved CR or CR with incomplete count recovery (CRi). One case experienced a relapse 117 days after the first CD38 CAR-T cell infusion and went into remission after the second infusion. All six of the patients had clinically manageable side effects, including grade I–II CRS and grade III hepatotoxicity. The results of the preliminary studies indicated that CD38 was a viable CAR-T target for the therapy of AML. And there are a number of clinical trials under recruitment (NCT05239689, NCT04351022).

## Targets that have entered pre-clinical trials

A number of new targets have entered preclinical trials in addition to those that have already been the subject of clinical trials. These targets, such as CD117, FRβ, CD93, TIM3, and others, are currently being examined in animal models to determine their efficacy and safety.

### CD117

CD117 (c-kit) plays an important role in HSC homeostasis [32, 112]. Its level of expression correlates with the prognosis of AML.

Preclinical research on CD117 is currently underway. Because CD117 is highly expressed in HSPC, CAR treatments targeting CD117 can cause severe suppression of hematopoiesis. Therefore, a bridge to transplantation is necessary. To this end, Myburgh et al. proposed a three-step immunotherapy technique consisting of the eradication of AML cells and original HSPCs via CD117 CAR-T cells, cessation of clearance effects, and finally, transplantation of healthy HSPC [32]. Complete elimination of healthy and leukemic cells was shown in a xenograft mouse model with CAR-T treatment, and subsequent hematopoiesis recovery after depletion of CAR-T cells with anti-thymocyte globulin and rituximab, followed by HSCT. Several in vitro investigations showed that, despite promising findings, low target antigen expression and loss of this antigen under selective therapeutic pressure can lead to CAR-T cells not fully exerting their effects [32]. Therefore, more investigation into the benefits and downsides of CD117 CAR-T cell therapy is required, along with an examination of how antigen loss can affect clinical trials in the future.

### CD4

CD4, a T cell membrane glycoprotein that binds to major histocompatibility complex class II antigens, is expressed at significantly high levels in some AML subtypes. For instance, CD4 is expressed in 65.0% and 78.3% of the acute myeloid leukemia and acute monocytic leukemia, respectively, but only in 30–40% of other subtypes. It is neither expressed on HSPCs, nor on non-hematopoietic cells. CD4 is a promising target for CAR-T treatment in AML because of its consistent, albeit low, expression.

The initial CD4 CAR treatment preclinical trial, assessing its safety and efficacy, was undertaken by Huda Salman et al. The result showed that it was effective at killing CD4 + AML cells in culture and in animals [112]. It inhibited the growth of leukemia cells in a xenograft mice model and improved survival time. Transient CD4 + T cell reduction and off-target toxicity were still detected since CD4 is also expressed in T cells; however, hematopoiesis was unaffected. These results provide credence to CD4 CAR-T cells’ feasibility as a bridging therapy preceding HSCT for tumor burden reduction or remission induction. Additionally, a safety switch based on alemtuzumab (anti-CD52 antibody) could be utilized to rapidly and completely reduce CD4 + CAR-T cell levels in patients who are ineligible for HSCT transplantation, optimizing killing efficacy whilst minimizing toxicity.

### FRβ

A total of four receptors—FRα, FRβ, FRγ, and FRδ—make up the folate receptor (FR) family and are found in varying degrees of expression throughout different organs and tissues. Epithelial cells are the only ones that express FRα, although myeloid hematopoietic cells predominately express FRβ. In addition, FRβ can also be induced to be up-regulated during macrophage activation, which is a possible mechanism for off-target toxicity of FRβ CAR-T cells [113].

Preclinical testing shows that FR CAR-T has anti-leukemic activity both in vitro and in vivo. However, the treatment’s potential efficacy in people with low FRβ expression is concerned, because the degree of CAR activation is correlated with the expression level of FR. Researchers have found that ATRA can increase FRβ expression in primary AML cells. Further stimulation of FR expression on AML in vitro was observed when ATRA was combined with histone deacetylase (HDAC) inhibitors [114]. Optimizing the use of ATRA in conjunction with other FR-inducing treatments, represented by HDAC, may not only extend the therapeutic reach of ATRA in AML, but also the efficacy of FRβ-targeted therapy.

FRβ CAR-T cell treatment has entered preclinical trials but not clinical trials.

### CD93

CD93 is a transmembrane antibody of the C-type lectin family that has a role in both cell–cell adhesion and host immune defense.

Richards et al. collaborated with Dr. Majeti to investigate a CD93 CAR-T preclinical trial in 2021 [63]. In vivo, the study showed that although damage to HSPCs was minimal, there was severe “on-target, off-tumor” injury due to the ubiquitous expression of CD93 on endothelial cells. The team developed a non-gated CAR-T design optimization strategy, as a result, allowing non-gated CAR T cells to avoid the off-tumor toxicity. These non-gated CD93 CAR T cells also expressed a second inhibitory CD19 CAR (iCAR), which was chosen for this investigation because it selectively targeted CD19, an antigen that is present on healthy cells but absent from AML cells. A CD19-specific inhibitory CAR consists of a CD19 scFv, a CD8α transmembrane signaling internal domain, and an immunoreceptor tyrosine-based inhibitory motif which encodes proteins, including PD-1 and TIGIT. This non-gated CAR-T demonstrated “on-target, on-tumor” cytotoxicity to CD93 + AML cells, while “on-target, off-tumor” effect to CD93 + immortalized human umbilical vein endothelial cells which was engineered to stably express truncated CD19, thus killing the AML cells but avoiding the toxicity to endothelial cell.

### MSLN

Mesothelin (MSLN), which is completely absent during normal hematopoiesis, is highly expressed in AML cells. This differential expression suggests a new target for CAR-T therapy with the goal of protecting hematopoiesis.

Preclinical testing of MSLN CAR-T for the treatment of AML was undertaken by Quy Le *et al* [115]. Primary AML cells and a CD34^+^CD38^−^ cell subpopulation were shown to express MSLN at the cell surface. To prove that MSLN is a therapeutically realistic target for CAR-T cell therapy in AML, the researchers used a xenograft model to show that MSLN CAR-T successfully killed MSLN^+^ AML cells while also targeting and eliminating CD34^+^CD38^−^ cells without interfering with the function of normal HSCs. Additionally, the team found that suppression of ADAM17 metalloproteinase, a protease that promotes MSLN shedding, could help to sustain CAR-T function.

### IL-10R

Interleukin receptor 10 receptor (IL-10R) includes two α and two β molecules.

Overexpression of IL-10R was found in the vast majority of AML cells, and IL-10 was found to promote cell proliferation in these cells by activating the IL-10R/PI3K/AKT/OCT4 signaling axis [116]. To take advantage of this, a new CAR-T cell was developed using IL-10 as its natural ligand as the antigen-binding structural domain; this CAR-T cell showed a considerable killing capability on AML cells both in vitro and in vivo. Additionally, the expression of IL-10 on the surface of CAR-T cells had no impact on their survival, biological activity, or ability to proliferate, and normal hematopoietic cells, like HSPCs, appeared to sustain no off-target damage. These findings imply that IL-10R is a promising AML CAR-T treatment target.

### WT1

Wilms Tumor 1 (WT1) is an intracellular protein, and a zinc-finger transcription factor frequently associated with cancer. It plays a role in many different biological processes, such as organ development, differentiation, proliferation, and apoptosis [59]. Its expression is considerably elevated in various types of solid tumors and hematological malignant cells but relatively modest in healthy tissues. A poorer prognosis is seen in AML when WT1 is overexpressed.

Intracellular localization of WT1 prevents antibodies and antibody-derived CAR-T cells from recognizing its antigens [60]. So, it is difficult in generating effective WT1 CAR-T cells. Researchers have discovered that peptides produced from intracellular WT1 can be expressed in the context of human leukocyte antigen on the surface of tumor cells (HLA). WT1 CAR can be designed based on the monoclonal antibody, such as ESK1 [60], which can recognize WT1/HLA A2 complexes. Moreover, if IL-12 gene is integrated into CAR, it can improve T-cell activity and amplify the therapeutic benefit. This approach was applied by Rafiq S. et al., who discovered that co-administration of IL-12 with T cell receptor-mimic CAR-T improves the efficiency of T cells against tumors and has the potential to boost anti-tumor treatment responses.

### GRP78 (HSPA5)

Glucose regulating protein 78 (GRP78) is typically found in the endoplasmic reticulum (ER) and plays a crucial role in controlling the highly conserved unfolded protein response during species evolution. Because the ER has so little space, GRP78 is shuttled out to the cell surface in a cancer-specific form. This process has been observed in many types of cancer, both solid and blood-related. Despite its potential as a target for CAR-T treatment in AML due to its expression properties, its efficacy has been the subject of relatively few investigations.

To determine whether or not GRP78 CAR-T cells could be used to treat AML, Nikhil Hebbar et al. conducted in vitro and in vivo tests, finding that the CAR-T cells successfully recognized and eradicated GRP78^+^ AML, but HSPCs did not elicit a substantial toxic response [117]. Blocking differentiation with dasatinib may also increase the target-killing impact of CAR-T because antigen-dependent T cell differentiation may limit CAR-efficacy.

## Future directions

For the therapy of AML, researchers are looking into a wide variety of avenues. Though some offer more promise than others, the development of any of them could lead to useful therapeutics. The biochemical pathways that these prospective targets cover need to be thoroughly explored in order to guarantee safe and effective therapeutics.

Transmembrane proteins during CAR exploration phase include the well-known programmed death 1 (PD1) protein. Despite the critical role played by the PD-1/PD-L1 axis in immunosuppression in AML, it is not known if PD-L1 is constitutively expressed in AML primary cells. The expression of PD-L1 may be upregulated in response to inflammatory stimuli such as toll-like receptor (TLR) ligands and interferon. A poorer prognosis may be associated with AML cells expressing PD-L1 and/or PD-L2 [64]. Monoclonal antibodies and blockers targeting PD-1 can effectively induce anti-tumor immune responses, reduce tumor burden, and increase OS in AML patients. Studies have shown that PD-1-deficient CLL-1 CAR-T cells are superior at killing off AML cells. The PD-1-deficient CLL-1 CAR-T cells were effective in treating two individuals with R/R AML, resulting in molecular complete remission and incomplete hematological recovery for both.

Just like WT1 mentioned above, unique intracellular tumor-specific mutational epitopes can also be employed for CAR-T cell treatment, and studies targeting intracellular antigens have the potential to expand CAR recognition beyond extracellular antigens. To do this, innovative strategies, including TCRm CAR, have been designed and are currently being successfully evaluated in scientific cases [60]. Studies on IDH1, IDH2, RHAMM, Survivin, hTERT, and many other topics have been published [118, 119]. Additionally, the sequentially tumor-selected antibody and antigen retrieval (STAR) system can be used to screen for nanobodies that preferentially bind to AML cells [97], and small conditional RNA (scRNA)-seq can provide information on immunotherapeutic target screening [120].

More cancer patients may benefit from CAR-T cell treatment if it is expanded to the targeting of intracellular antigens.

## Conclusions

The treatment of AML includes traditional chemotherapy, targeted drug and hematopoietic stem cell transplantation, as well as CAR-T and CAR-NK cell therapies. Every therapeutic way has its unique advantages and challenges.

Traditional chemotherapy regimens for AML have been developed so mature until now and are the first-line treatment for newly diagnosed AML. While the organ damage and hematopoietic inhibition caused by chemotherapeutics may be fatal to patients, especially to the elders. Novel small molecular targeting drugs, such as gilteritinib (FLT3 inhibitor) [3], enasidenib (IDH2 inhibitor) [4], ivosidenib (IDH1 inhibitor) [5], or venetoclax (BCL-2 inhibitor) [6], have shown promising results. However, the cost of these drugs can be a challenge, and they may not be accessible to all patients. Stem cell transplantation remains a viable option for AML, particularly in patients who are young and have a suitable donor. However, this treatment can also cause severe side effects, such as graft-versus-host disease and infection. CAR-T and CAR-NK cell therapies have shown promising results in the treatment of AML, particularly in patients who are relapsed or refractory after standard therapies. CAR-T and CAR-NK cell therapies can target specific antigens on cancer cells and kill the tumor. CAR-NK cell therapy has the potential to target a wider range of tumor cells, including those that are resistant to T cell therapy. However, the use of CAR-T cell therapy can also cause severe side effects, such as cytokine release syndrome and neurotoxicity, which can be life-threatening. Furthermore, the effectiveness of CAR-NK cell therapy may be limited by the availability of suitable NK cells for engineering.

The treatment approach for AML should be tailored to the individual patient’s condition and disease severity. For the younger, or those in good physical condition, traditional chemotherapy followed by stem cell transplantation may be the preferred treatment option, as it can provide long-term therapeutic benefits. For the elder, or those with comorbidities, targeted drug therapy may be the more suitable option, as it can offer a gentler treatment and can be administered at home. For the patients who have already undergone traditional treatments, but have not achieved sustained remission, CAR-T and CAR-NK cell therapies may be the best option.

In general, the treatment sequence for AML should be determined based on the individual patient’s condition and disease severity. Stem cell transplantation following chemotherapy may be the first-line treatment option for the younger or fit patients. Targeted drug therapy may be a more suitable option for older or frail patients. CAR-T and CAR-NK cell therapies may be the best option for patients with relapsed and/or refractory AML. When selecting a treatment approach, besides individual patient’s condition and comorbidities, the communication and collaboration with their family are also important.

## Data Availability

Not applicable.

## Abbreviations

Allo-HSCT: Allogenic hematopoietic stem cell transplantation
AML: Acute myeloid leukemia
ATRA: All-trans retinoic acid
CAR-T: Chimeric antigen receptor T cell
CLL-1: C-type lectin-like molecule-1
CR: Complete remission
CRS: Cytokine release syndrome
ER: Endoplasmic reticulum
FDA: U.S. Food and Drug Administration
FLT3: Fms-like tyrosine kinase 3
FLT3Lg: FLT3 ligament
FR: Folate Receptor
GRP78: Glucose regulated protein 78
GVHD: Graft-versus-host disease
HDAC: Histone deacetylase
HSPC: Hematopoietic stem and progenitor cells
IL: Interleukin; ITD: Internal tandem duplication
KDEL: Lys-Asp-Glu-Leu, a peptide sequence
LILRB: Leukocyte immunoglobulin-like receptor B
LSC: Leukemic stem cells
mAb: Monoclonal antibodies
MDS: Myelodysplastic syndromes
MLFS: Morphological leukemia-free state
MM: Multiple myeloma
MSLN: Mesothelin
NK: Natural killer cell
NKG2DL: Natural killer group 2, member D ligands
OS: Overall survival
PD1: Programmed cell death protein 1
PI3K: Phosphoinositide 3-kinases
R/R: Relapsed or refractory
RevCAR: Reversible chimeric antigen receptor
scFv: Single-chain variable fragment
SUPRA: Split, universal, and programmable CAR
TCRm: T-cell receptor mimic
TKD: Tyrosine kinase domain
TM: Targeting module
TNF: Tumor necrosis factor
Treg: Regulatory T cells
UniCAR: Universal CAR
5’-AZA: 5’-Azacytidine
